# Exploring type 2 inflammation and disease complexity: perspectives of a clinical immunologist

**DOI:** 10.3389/fimmu.2026.1872479

**Published:** 2026-09-03

**Authors:** Yuval Tal, Limor Rubin, Ariel Munitz, Irit Adini

**Affiliations:** 1 Allergy and Clinical Immunology Unit, Department of Medicine, Hadassah, University Medical Center, Jerusalem, Israel; 2 Department of Clinical Microbiology and Immunology, Faculty of Health and Medical Sciences, Tel Aviv University, Tel Aviv, Israel; 3 Department of Surgery, Center for Engineering in Medicine and Surgery, Harvard Medical School, Boston, MA, United States

**Keywords:** allergy, asthma, atopic dermatitis, TSLP, type 2 inflammation

## Abstract

Type 2 inflammatory diseases are often grouped together as a single immunological entity, driven by shared cytokines and overlapping pathways. This framework has led to the development of biologic therapies targeting upstream epithelial-derived “alarmins”, including thymic stromal lymphopoietin (TSLP), interleukin (IL)-33, and interleukin (IL)-25. These mediators promote downstream cytokine responses, most notably IL-4, IL-5, and IL-13, which orchestrate type 2 inflammation across multiple tissues. Despite these shared molecular features, type 2-associated diseases exhibit substantial clinical heterogeneity and variable responses to targeted therapies. A notable example is TSLP blockade, which has shown significant clinical efficacy in asthma but failed to demonstrate similar benefit in atopic dermatitis. From a clinical immunology perspective, we explore the complexity of type 2 inflammation. We discuss the dual pro- and anti-inflammatory roles of TSLP isoforms (long form and short form), the contribution of compensatory inflammatory pathways, and the impact of the tissue microenvironment on disease-specific immune responses. These factors may underlie divergent therapeutic outcomes despite targeting a shared upstream mediator. A better understanding of this complexity may help explain the limitations of a linear, cytokine-centered model of type 2 inflammation and support the development of more precise therapeutic strategies, including targeting shared downstream signaling components and combination approaches.

## Introduction

Type 2 inflammation is a complex and multifaceted immune response, involving both innate and adaptive immune cells, including type 2 innate lymphoid cells (ILC2s), dendritic cells (DCs), T helper 2 (Th2) cells, basophils, eosinophils, and mast cells. Triggered by environmental and genetic factors, these cells release a network of cytokines, most notably interleukin (IL)-4, IL-5, and IL-13, which orchestrate type 2 inflammation through immunoglobulin E (IgE) production, eosinophil recruitment, Th2 differentiation, M2 macrophage polarization, goblet cell hyperplasia, mucus hypersecretion, and epithelial barrier disruption (1).

**Figure 1:**
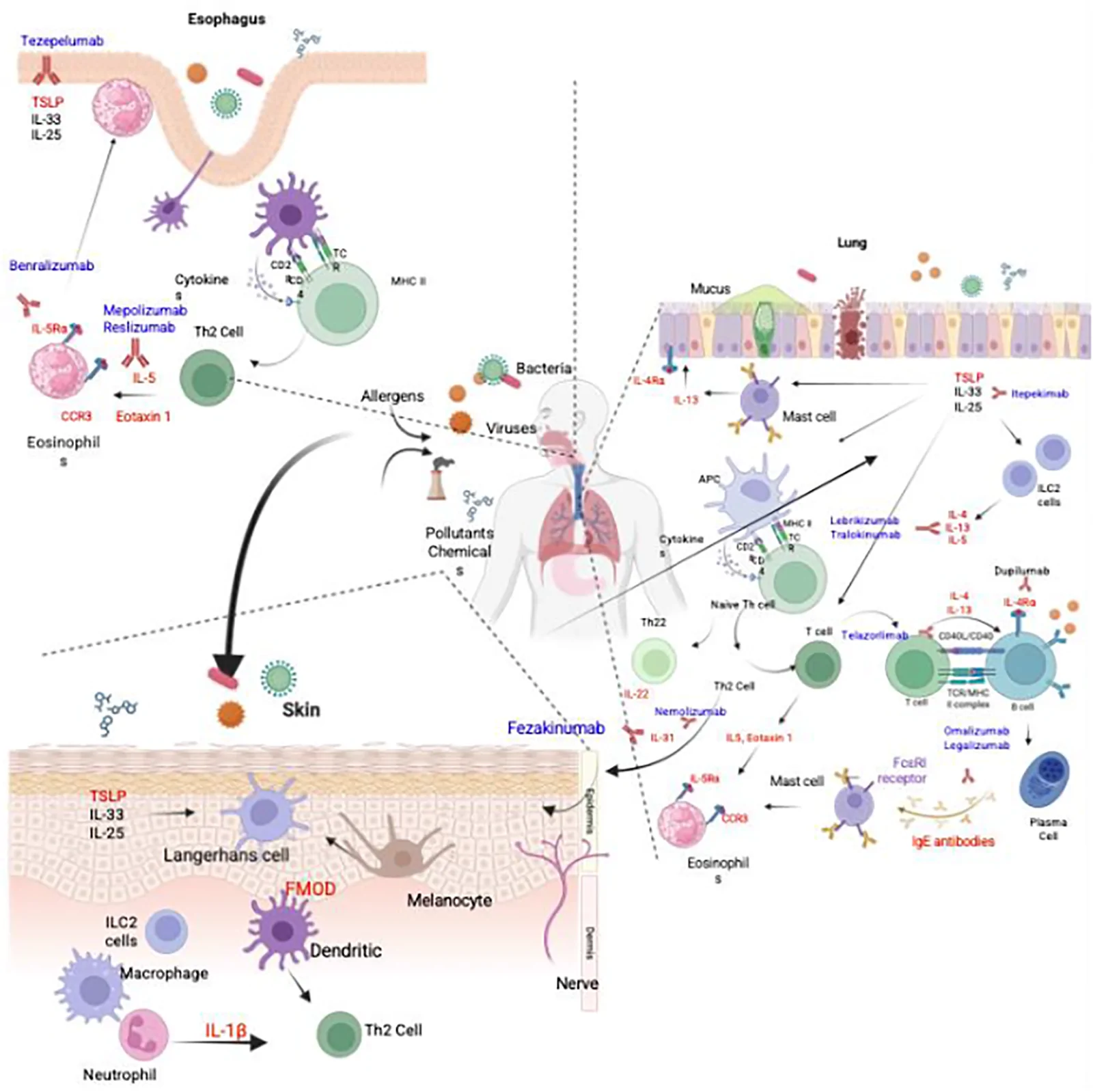
Type 2 inflammatory pathways and therapeutic targets across barrier tissues. The schematic highlights shared type 2 immune mechanisms and tissue-specific inflammatory responses across the esophagus, lung, and skin. Environmental triggers act on and disrupt epithelial barriers, leading to the release of epithelial-derived alarmins, including TSLP, IL-33, and IL-25. These signals promote activation of dendritic cells, Langerhans cells, mast cells, ILC2s, Th2 cells, eosinophils, B cells, and other tissue-resident cells, resulting in the production of cytokines such as IL-4, IL-5, IL-13, IL-31, and other tissue-dependent mediators. Tissue-specific biologic therapies and their targets are shown in blue, including tezepelumab/TSLP, benralizumab/IL-5Rα, mepolizumab and reslizumab/IL-5, itepekimab/IL-33, dupilumab/IL-4Rα, lebrikizumab and tralokinumab/IL-13, nemolizumab/IL-31Rα, omalizumab and ligelizumab/IgE, telazorlimab/CD40–CD40L costimulatory signaling, and fezakinumab/IL-22.

This inflammatory axis often involves upstream epithelial-derived “alarmins”, including thymic stromal lymphopoietin (TSLP), IL-33, and IL-25, which are released following epithelial injury or exposure to allergens, pollutants, or pathogens (2). These mediators promote further Th2 skewing and contribute to a dynamic, self-amplifying inflammatory process, in which epithelial damage both initiates and sustains the immune response (**Figure 1**).

Despite this shared molecular framework, type 2-associated diseases frequently demonstrate marked clinical heterogeneity and variable responses to targeted therapies. While biologic agents directed against key cytokines or upstream mediators have transformed the treatment landscape, their efficacy remains inconsistent across different tissues and diseases.

This raises a central question: why does targeting a major upstream cytokine such as TSLP result in significant clinical benefit in asthma, yet fail to produce comparable effects in atopic dermatitis?

In this perspective, we revisit type 2 inflammation from a clinical immunology standpoint, aiming to highlight its non-linear and context-dependent nature. We focus on the role of TSLP isoforms, compensatory inflammatory pathways, and the influence of the tissue microenvironment, as potential explanations for divergent therapeutic outcomes despite targeting a shared upstream mediator.

## The complex role of epithelial cell-derived cytokines in type 2 inflammation

Epithelial cells serve as both a physical barrier and an active immunological sentry. Upon exposure to viral, fungal, allergenic, or pollutant-induced insults, the damaged epithelium releases upstream “alarmins”, including thymic stromal lymphopoietin (TSLP), interleukin (IL)-33, and IL-25. These mediators initiate and amplify type 2 inflammation through distinct yet often overlapping mechanisms.

Among these, TSLP has emerged as a central upstream regulator of type 2 inflammation. It is a pleiotropic cytokine predominantly derived from epithelial cells, acting on multiple immune cell lineages via a high-affinity receptor complex composed of TSLPR and the IL-7 receptor α-chain (3). Although sequence identity varies across species, its functional activity is conserved between mice and humans (4). Following epithelial barrier disruption, TSLP is among the earliest cytokines released and contributes to the pathogenesis of asthma, atopic dermatitis (AD), allergic rhinitis, and eosinophilic esophagitis (EoE) (3, 5). Mechanistically, TSLP upregulates co-stimulatory molecules on dendritic cells (DCs), thereby promoting T helper 2 (Th2) polarization (6, 7), while also enhancing cytokine and chemokine release from mast cells, innate lymphoid cells (ILCs), and macrophages (8–12).

Despite this central role, the clinical relevance of TSLP appears to differ across diseases. While TSLP appears to be a major upstream driver in asthma, the inflammatory network in CRSwNP may involve a relatively broader contribution from other alarmins, including IL-33 and IL-25 (13, 14). Consistent with its upstream position, TSLP blockade has demonstrated significant clinical efficacy in asthma, leading to the approval of the anti-TSLP monoclonal antibody tezepelumab (15). However, despite increased TSLP expression in AD, clinical trials with tezepelumab have failed to demonstrate similar benefit, suggesting that the role of TSLP is more context-dependent than initially appreciated (16, 17).

A key factor that may underlie these differences is the existence of two distinct TSLP isoforms: the long form (lfTSLP) and the short form (sfTSLP). The long isoform is primarily pro-inflammatory, with tightly regulated expression that is upregulated in response to Toll-like receptor (TLR) signaling, including TLR3 ligands such as double-stranded RNA or synthetic analogs (e.g., poly I:C), as well as TLR2 and TLR6 pathways (18). lfTSLP signals through the TSLPR/IL-7Rα receptor complex, leading to activation of downstream transcriptional programs, including upregulation of OX40 ligand (OX40L) on DCs and subsequent Th2 differentiation (7). Therapeutically, this pathway is further supported by ongoing phase 3 studies targeting OX40L in AD (7, 19, 20).

In contrast, sfTSLP appears to serve constitutive “housekeeping” and antimicrobial functions (21, 22). It promotes phosphorylation of p38 MAPK and ERK1/2, with minimal activation of STAT5, and its receptor remains unidentified (23, 24). Importantly, sfTSLP is functionally distinct and may act, at least in part, as an antagonist to lfTSLP (25, 26).

**Figure 2:**
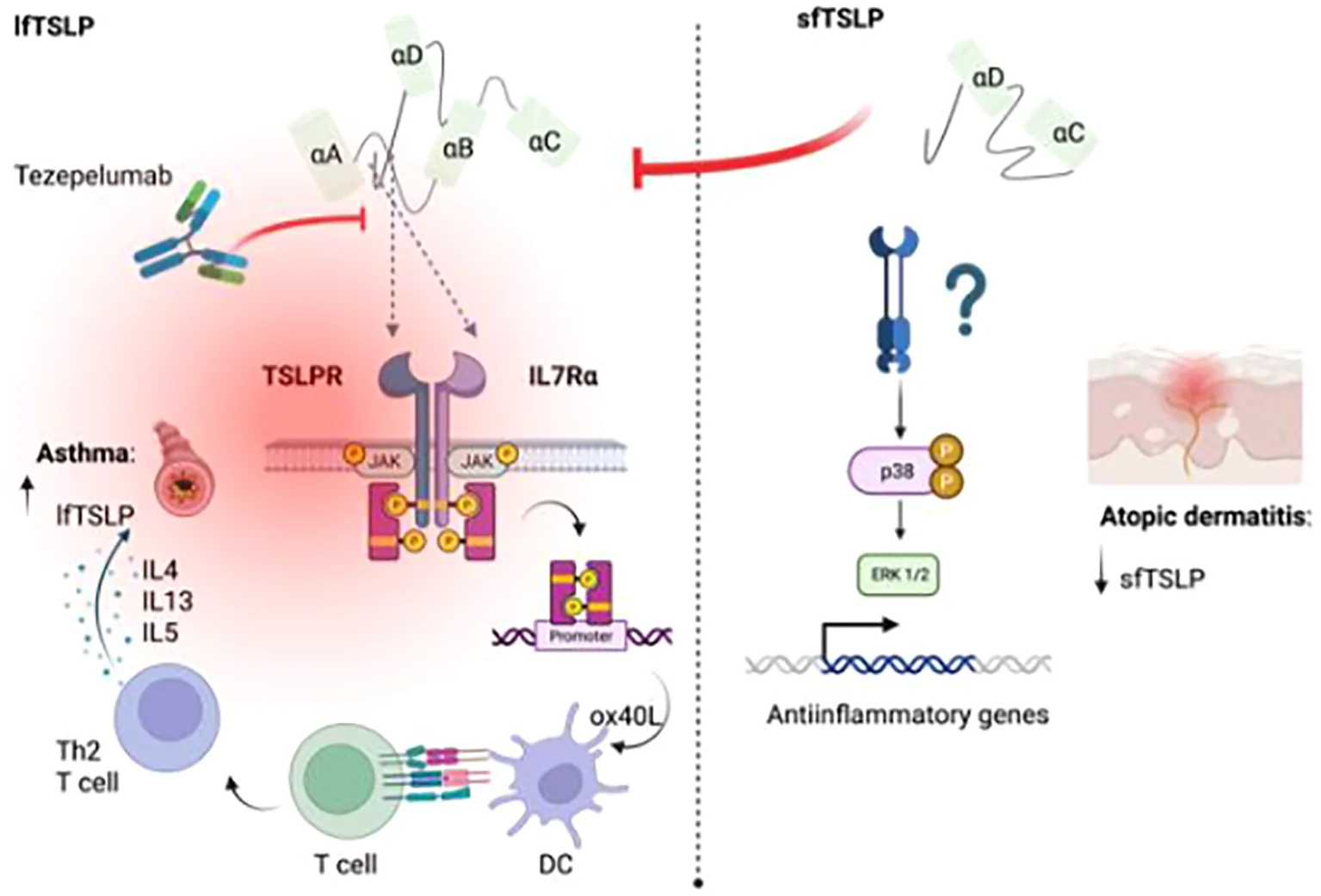
Distinct roles of long-form and short-form TSLP in type 2 inflammation. The schematic illustrates increased long-form TSLP (lfTSLP) in asthma and reduced short-form TSLP (sfTSLP) in atopic dermatitis, based on available data. lfTSLP signals through the TSLPR/IL-7Rα receptor complex, activating JAK/STAT-dependent pathways that promote dendritic cell activation, OX40L expression, Th2 polarization, and the production of type 2 cytokines, including IL-4, IL-5, and IL-13. Tezepelumab inhibits lfTSLP by preventing its interaction with the TSLPR/IL-7Rα receptor complex. In contrast, sfTSLP is proposed to have homeostatic and potentially anti-inflammatory functions, possibly involving p38 and ERK1/2 signaling pathways.

These differences between isoforms may have direct clinical implications. Tezepelumab selectively targets lfTSLP by binding to a specific three-dimensional conformation between its αD and αA helical regions. In asthma, where lfTSLP levels are markedly increased, the expression and regulation of sfTSLP remain insufficiently characterized (27), this approach is effective. In contrast, in AD, sfTSLP expression is reduced and lfTSLP is only modestly elevated, which may limit the therapeutic impact of selective lfTSLP blockade (**Figure 2**).

Although these observations raise the possibility that differences in TSLP isoform expression contribute to disease-specific therapeutic responses, this interpretation remains hypothetical. The existence of distinct long-form and short-form TSLP isoforms, their differential expression patterns, and their potentially divergent biological activities are supported by published studies. However, direct evidence that isoform balance explains variable clinical responses to TSLP blockade across asthma, atopic dermatitis, and other type 2 inflammatory diseases is still limited. Therefore, TSLP isoform biology should therefore be viewed as a plausible mechanistic framework that requires further validation rather than as an established explanation for therapeutic outcomes (24).

## Clinical implications of variable TSLP dependence

Clinically, the variable efficacy of TSLP blockade across type 2 inflammatory diseases support the concept that TSLP is not uniformly dominant across all tissue contexts. In severe asthma, where epithelial alarmin activity and long-form TSLP expression are strongly implicated in airway inflammation, tezepelumab has demonstrated robust clinical benefit, including reductions in exacerbations and improvement in lung function. In contrast, in atopic dermatitis, clinical responses to anti-TSLP therapy have been more modest, suggesting that cutaneous inflammation is maintained by redundant or compensatory pathways beyond TSLP alone. Similarly, in eosinophilic esophagitis while type 2 inflammation is a key driver of disease pathology, clinical experience with upstream cytokine blockade remains limited, and symptom improvement may not directly parallel eosinophil suppression, likely reflecting contributions from tissue remodeling, epithelial barrier dysfunction, and parallel cytokine networks (28).

## Compensatory mechanisms and additional inflammatory pathways

In atopic dermatitis (AD), blocking TSLP alone may not be sufficient, as multiple cellular and molecular mechanisms can bypass its activity. For example, Basophils, key effector cells in type 2 responses, for example, can be activated independently of TSLP through alternative pathways such as interleukin (IL)-3 signaling. In parallel, negative feedback mechanisms may further modulate the relative contribution of TSLP. IL-37, an anti-inflammatory cytokine of the IL-1 family, has been shown to downregulate both TSLP expression and basophil activation.

Interestingly, these regulatory pathways do not behave uniformly across diseases. In conditions characterized by episodic flares, such as asthma, IL-37 levels tend to vary inversely with disease activity (29). In contrast, in chronic inflammatory states such as severe AD, IL-37 levels may remain persistently elevated (30), potentially reducing the relative contribution of TSLP to the overall inflammatory process. Even when TSLP is present, its functional impact may therefore be attenuated by parallel or counter-regulatory pathways, which could partly explain the limited effectiveness of TSLP-targeted therapies in certain settings.

This concept is not restricted to individual cytokines and challenges the classical linear view of type 2 inflammation. Certain immunodeficiencies illustrate how similar clinical phenotypes can arise through distinct immunological pathways. Patients with common variable immunodeficiency (CVID), who often have low or absent immunoglobulin E (IgE), can still present with clinical features typically associated with IgE-mediated diseases, such as allergic rhinitis. This apparent paradox suggests that similar immunologic end states may be reached through alternative, non-IgE-dependent pathways, potentially involving IL-4 and IL-13 signaling (31).

Taken together, these observations point toward a model in which type 2 inflammatory responses are not driven by a single dominant pathway, but instead arise from a network of interacting and partially redundant mechanisms. In such a system, multiple routes can lead to similar clinical outcomes, which may explain why targeting a single upstream mediator, such as TSLP, results in variable therapeutic responses across diseases. From a clinical perspective, this may help explain why blocking an upstream mediator like TSLP can be highly effective in TSLP-dominant endotype, such as some forms of severe asthma. In contrast, TSLP inhibition is likely to be less effective in diseases or disease stages where aother pathway, for example IL-33, IL-4/IL-13, IL-5, IL-1β, barrier dysfunction, or tissue remodeling, become dominant drivers.

## TSLP and corticosteroid responsiveness

Corticosteroids remain the cornerstone of therapy for allergic and type 2 inflammatory diseases; however, responses vary across patients and disease endotypes. Emerging evidence suggests that epithelial-derived TSLP may contribute to corticosteroid-insensitive inflammation by sustaining dendritic cell activation and reinforcing downstream type 2 immune circuits that are not fully suppressed by glucocorticoid signaling. In particular, TSLP activates STAT5dependent survival pathways, including BclxL, enabling type 2 inflammatory cells to survive and function despite corticosteroid exposure. In airway disease, higher TSLP levels have been linked to steroid resistant ILC2 responses, and in some models blocking TSLP related signaling restores corticosteroid sensitivity (32).

More broadly, corticosteroid resistance is increasingly recognized as a multifactorial process involving impaired glucocorticoid receptor signaling, altered chromatin remodeling (including histone deacetylase activity), and persistent inflammatory amplification loops driven by epithelial alarmins and type 2 cytokines. In this context, TSLP likely acts as an upstream regulator that can sustain inflammatory signaling despite corticosteroid treatment in certain settings, although it may also be partially suppressed by glucocorticoids depending on tissue and disease context. Accordingly, the relationship between TSLP signaling and corticosteroid responsiveness is complex and context-dependent. These considerations support the view that TSLP is one component of a broader epithelial–immune network that shapes inflammatory persistence and treatment sensitivity across type 2 inflammatory diseases.

## TSLP secretion in atopic dermatitis and the role of innate immune cells

In atopic dermatitis (AD), the contribution of epithelial-derived TSLP appears to vary with disease depth and severity. In more superficial, milder forms of AD, TSLP secretion from epithelial cells is thought to play a central role in initiating and maintaining the type 2 response. However, as inflammation extends into deeper skin layers in more severe disease, its relative importance seems to diminish (33).

In these deeper compartments, innate immune cells such as neutrophils and macrophages become more prominent contributors to the inflammatory process. These cells secrete interleukin (IL)-1β, a key cytokine associated with type 1 inflammation and a major product of inflammasome activation. IL-1β can activate already primed T helper 2 (Th2) cells toward a type 2 response, for example through enhanced IL-13 secretion, but does so via pathways that are largely independent of TSLP.

This shift in the dominant inflammatory drivers may allow type 2 responses to persist despite effective upstream blockade of TSLP, providing a potential explanation for the limited efficacy of anti-TSLP therapies in severe AD (33, 34).

## IL-33: an additional alarmin

IL-33 represents another epithelial-derived “alarmin” that contributes to type 2 inflammation, but through mechanisms that only partially overlap with those of TSLP. It is a member of the interleukin (IL)-1 cytokine family and, unlike many secreted cytokines, lacks a classical signal sequence (35). Instead, IL-33 is constitutively expressed in the nuclei of epithelial and endothelial cells, as well as in fibroblasts and certain immune cells such as macrophages (35–37).

Following cellular damage or necrosis, IL-33 is released into the extracellular environment, where it can be processed and activated by proteolytic cleavage (38, 39). It signals through a receptor complex composed of IL1RL1 (ST2), which is specific for IL-33, and the shared IL-1 receptor accessory protein (IL-1RAcP) (40). This receptor is expressed on multiple immune cell types, including mast cells (MCs), eosinophils, type 2 innate lymphoid cells (ILC2s), T helper 2 (Th2) cells, and regulatory T cells (41, 42).

Activation of these cells by IL-33 promotes the production of key type 2 cytokines, particularly IL-5 and IL-13, leading to eosinophilic inflammation, mucus hypersecretion, and airway hyperresponsiveness. In experimental models, overexpression of IL-33 in the skin or esophagus induces atopic dermatitis, and eosinophilic esophagitis (EoE)-like phenotypes (43–45).

While IL-33 can clearly drive type 2 inflammation in multiple tissues, its relative contribution appears to vary across diseases. In asthma, for example, targeting the IL-33/ST2 axis has shown largely supportive results in clinical development, suggesting a meaningful, though not exclusive, role within a broader network of upstream mediators (46). This raises the possibility that, similar to TSLP, the functional role of IL-33 may differ between airway and skin inflammation (47), although this remains less clearly defined.

## Shared and unique activities of effector cytokines in type 2 inflammation

Type 2 inflammation underlies multiple chronic inflammatory diseases, including asthma, atopic dermatitis (AD), chronic rhinosinusitis with nasal polyps (CRSwNP), and eosinophilic esophagitis (EoE). These conditions share common molecular features, including a conserved type 2 gene signature (NCBI Gene Expression Omnibus: GSE58640), and often co-occur within the same individuals as part of the “atopic march” (48). Epidemiologically, this overlap is striking; for example, up to 80% of patients with asthma also suffer from allergic rhinitis (49).

Despite this shared framework, clinical outcomes and responses to targeted therapies differ substantially, suggesting that the downstream effector pathways are not interchangeable. The hallmark type 2 cytokines: IL-4, IL-5, and IL-13, play central roles in orchestrating the inflammatory response, yet their relative contributions and functional effects vary across tissues and diseases.

The IL-4/IL-13 axis provides a clear example of this complexity. Both cytokines signal through receptor systems that share the IL-4 receptor α-chain (IL-4Rα), but differ in their composition and downstream effects. IL-4 can signal through the type 1 IL-4 receptor, composed of IL-4Rα and the common γ chain (γc), as well as through the type 2 receptor, which includes IL-4Rα and IL-13 receptor α1 (IL-13Rα1) (50–54). IL-13 primarily signals through receptor complexes involving IL-4Rα, IL-13Rα1, and IL-13Rα2 (51, 52). While IL-13Rα2 was initially considered a decoy receptor (55, 56), accumulating evidence suggests that it can also mediate active signaling (39, 52, 57). In addition, IL-13 may signal through alternative complexes that do not require IL-4Rα (58).

This receptor-level complexity translates into variable biological and clinical effects. Targeting IL-4 or IL-13 individually in asthma has often failed, likely due to compensatory signaling pathways (59). In contrast, selective IL-13 blockade with lebrikizumab has demonstrated clinical efficacy in atopic dermatitis (60). Similarly, differential roles of these cytokines are observed across tissues: in experimental models of asthma and AD, eosinophilic infiltration is primarily mediated by IL-4 signaling through the type 1 IL-4 receptor (51, 61, 62), whereas in EoE, IL-13 appears to play a dominant role via the type 2 receptor (63).

Additional examples further highlight this divergence. Antibodies targeting IL-5 or IL-5 receptor α demonstrate clear clinical benefit in asthma. However, they have thus far failed to show similar efficacy in EoE, despite effectively reducing eosinophil counts (64). This disconnects between biological effect and clinical outcome underscores the importance of context in determining therapeutic response.

Taken together, these observations suggest that shared molecular pathways do not necessarily translate into uniform disease behavior. Instead, tissue-specific factors and local immune networks shape how these cytokines function in different settings. This is further supported by disease-specific features, such as fibrin deposition in nasal polyps, which reflect distinct pathophysiological mechanisms (65).

While proof-of-concept evidence supports the effectiveness of targeting effector cytokines in type 2 inflammation, the optimal therapeutic strategy remains uncertain. One approach to address this complexity is the classification of diseases into distinct “endotypes,” as has been proposed for chronic rhinosinusitis and asthma (66, 67). Stratifying patients based on these underlying mechanisms may ultimately guide more precise and effective, endotype-driven therapeutic interventions.

## Type 2 inflammation - the effect of the microenvironment

The behavior of immune cells in type 2 inflammation is highly dependent on their surrounding microenvironment. Rather than acting in a fixed manner, the same cell can adopt different, and sometimes opposing, functions depending on local signals.

The Local Immunity and/or Remodeling/Repair (LIAR) hypothesis provides a useful framework for this concept. Eosinophils, for example, are not inherently pro-inflammatory. In a neutral environment, they participate in physiological processes such as mammary gland development, wound healing, and possibly the remodeling of the pubic symphysis before delivery. In contrast, within a type 2 inflammatory setting, they acquire a pro-inflammatory role, contributing to asthma, allergic diseases, and parasitic responses. Under different conditions, such as a type 1/17-polarized environment, eosinophils may even exert suppressive effects, including the induction of apoptosis, as demonstrated by Lee et al. (68).

This context-dependent behavior is not limited to circulating immune cells. Tissue-resident cells can further shape the local immune response. The skin provides a clear example. As an active immune organ, it exhibits distinct regulatory features, including melanocyte involvement in immune modulation. As shown by Adini et al. (69), melanocytes secrete fibromodulin, a small leucine-rich extracellular matrix protein that can inhibit dendritic cell (DC) maturation and activation. Differences in fibromodulin secretion between populations may influence immune activation, with lower levels associated with enhanced DC activation and potentially contributing to the increased prevalence of atopic disorders, asthma, systemic lupus erythematosus, and sepsis-related mortality observed in Black American populations (70).

Together, these observations emphasize that type 2 inflammation is shaped not only by cytokines, but also by the local cellular and tissue context in which these signals operate. This may further explain why targeting a single pathway yields variable outcomes across different organs and patient populations.

## Blurring the lines between traditional classifications

The traditional division of immune responses into type 1 and type 2 pathways is, in many ways, an oversimplification. It largely reflects historical definitions based on cytokine profiles, rather than the dynamic behavior of the immune system *in-vivo*.

Clinical observations increasingly suggest that these pathways are not independent, but rather interconnected and capable of shifting under therapeutic pressure. One example is Job syndrome (autosomal dominant hyper-IgE syndrome), caused by STAT3 loss of function. In this setting, impaired Th17 responses are accompanied by a compensatory skewing toward Th2 immunity. As a result, patients exhibit a combination of immunodeficiency and type 2-like manifestations, including dermatitis, elevated IgE levels, and eosinophilia, closely resembling atopic dermatitis at both the clinical and molecular levels.

Targeted interventions can further expose this plasticity. Treatment with dupilumab, an anti-IL-4Rα antibody, suppresses IL-4/IL-13 signaling and improves type 2-driven disease manifestations (71). At the same time, dampening Th2 activity may unmask or enhance alternative inflammatory pathways, including Th1- or Th17-associated responses. This is reflected in reported adverse events such as vitiligo, alopecia areata, psoriasis, and arthritis (72).

At the same time, immune modulation does not always result in a simple shift from one axis to another. There is also evidence that dupilumab can promote regulatory pathways, including Treg differentiation through increased Foxp3 transcription (73), suggesting a more complex rebalancing of the immune system rather than a binary switch.

Taken together, these observations challenge the notion of fixed immune “types” and instead support a more dynamic view of immune regulation, in which therapeutic interventions can reshape the balance between interconnected pathways in unpredictable ways.

## Toward personalized and multi-targeted therapies

As the complexity of type 2 inflammation becomes more apparent in clinical practice, it is increasingly clear that targeting a single cytokine is often insufficient. Instead, therapeutic strategies are shifting toward broader or more flexible approaches that account for overlapping and compensatory pathways.

One such approach is the targeting of shared downstream signaling components. Agents such as Janus kinase (JAK) inhibitors, which interfere with multiple cytokine pathways simultaneously, have shown clinical benefit in patients with atopic dermatitis (AD) who do not respond adequately to IL-4R blockade.

Combination strategies offer another way to approach this complexity. In selected patients with overlapping disease mechanisms, combining biologic agents, such as omalizumab with type 2-targeted therapies (e.g., anti-IL-5) or even with type 1-directed agents (e.g., anti-tumor necrosis factor), may provide a more effective way to control complex inflammatory networks (74, 75).

Emerging therapies further expand this concept. Nanobodies, for example, are engineered single-domain antibodies capable of simultaneously targeting multiple molecules. In parallel, agents directed against costimulatory pathways such as OX40/OX40L offer a broader immunomodulatory approach (76, 77).

Taken together, these strategies reflect a shift away from single-pathway inhibition toward a more integrated, context-dependent approach to treatment. The goal is no longer to identify one dominant cytokine, but to match the therapeutic strategy to the network of pathways driving disease in each patient.

## Conclusion

Type 2 inflammatory diseases share common molecular and cellular features, including epithelial barrier dysfunction and Th2-associated cytokine activity. At the same time, they differ in key drivers, upstream mediators, and the degree of redundancy within their inflammatory networks. These differences are not limited to distinct diseases such as asthma, chronic rhinosinusitis with nasal polyps (CRSwNP), atopic dermatitis (AD), and eosinophilic esophagitis (EoE), but extend across patients, tissues, and even different stages of the same disease.

Throughout this work, we have highlighted how these variations translate into divergent clinical outcomes, particularly in the context of targeted therapies. The example of TSLP blockade illustrates this well: a pathway that appears central in one disease may play a more limited or context-dependent role in another.

While proof-of-concept evidence supports the effectiveness of targeting type 2 cytokines, the optimal therapeutic strategy remains uncertain. Rather than focusing on a single dominant pathway, a more useful approach may be to consider the underlying network of interacting mechanisms in each clinical setting.

One practical step in this direction is the classification of type 2-associated diseases into distinct “endotypes, “ as proposed for chronic rhinosinusitis and asthma (66, 67). Stratifying patients based on these mechanisms may help guide more precise, context-dependent therapeutic decisions.

## Data Availability

The original contributions presented in the study are included in the article/supplementary material. Further inquiries can be directed to the corresponding authors.
